# Wine Lees as a Source of Antioxidant Compounds

**DOI:** 10.3390/antiox8020045

**Published:** 2019-02-16

**Authors:** María José Jara-Palacios

**Affiliations:** 1 Instituto de Investigaciones Vitivinícolas y Agroalimentarias (IVAGRO), Universidad de Cádiz, Campus de Puerto Real, 11510 Cádiz, Spain; mjara@us.es; 2 Food Colour and Quality Laboratory, Universidad de Sevilla, Facultad de Farmacia, 41012 Sevilla, Spain; 3 Department of Analytical Chemistry, Universidad de Sevilla, Facultad de Farmacia, 41012 Sevilla, Spain

**Keywords:** wine lees, phenolic compounds, antioxidant activity, winemaking byproducts

## Abstract

The winemaking industry produces large amount of byproducts, including grape pomace, stalks, and lees. Wine lees are a natural source of phenolic compounds, which have important antioxidant and biological properties. Due to the high quantities produced worldwide, this byproduct can be an ideal raw material for obtaining phenolic compounds that could be of interest in the food and pharmaceutical industries. In this mini review, the main characteristics of wine lees as well as their phenolic composition and antioxidant activity have been summarized from the information in the literature.

## 1. Introduction

Wine production is an important activity throughout the world. According to the International Organization of Vine and Wine (OIV) [1], 246.7 million hL of wine were produced in 2017, with the main wine-producing countries being Italy (39.3 million hL), France (36.7 million hL), and Spain (33.5 million hL).

The winemaking industry produces large amount of byproducts, including grape pomace, stalks and lees. These winemaking byproducts are considered as an important source of bioactive compounds with antioxidant activity, such as phenolic compounds [2]. Phenolic compounds are highly valued because they can be used for the pharmaceutical, cosmetic, and food industries.

Grape pomace and stalks have been widely studied in relation to its composition and bioactivity [3,4,5,6,7]. However, wine lees are the least studied and exploited byproducts from the wine industry. Therefore, the aim of this study is to collect information from literature about wine lees and their potential as a source of antioxidant compounds.

## 2. Wine Lees

According to literature and EEC regulation No. 337/79, wine lees can be defined as “a residue that is formed at the bottom of wine containers, after fermentation, during storage or after treatments, as well as the residue obtained after the filtration or centrifugation of this product” [8]. On the one hand, wine lees can be classified into three groups depending on the stage of vinification: first- and second-fermentation lees, which are formed during the alcoholic and malolactic fermentations, respectively, and aging wine lees formed during wine aging in wood barrels [9]. On the other hand, wine lees also can be classified depending on the particle size: heavy lees (between 100 μm and 2 mm, settling within 24 h) and light lees (<100 μm, between 1 and 24 μm, and in suspension at least 24 h after agitation) [10,11].

The main characteristics of wine lees are acidic pH (between 3 and 6), a chemical oxygen demand above 30,000 mg/L, potassium levels around 2500 mg/L, and phenolic compounds in amounts up to 1000 mg/L [12].

This winemaking byproduct is composed of solid and liquid fractions [13]. The solid fraction is a combination of yeasts, organic acids (mainly tartaric acid), insoluble carbohydrates (such as cellulosic or hemicellulosic materials), inorganic salts, lignin, proteins, phenolic compounds, and pulp and other parts of the grape. The liquid fraction is mainly composed of ethanol and organic acids, as lactic acid and acetic acid [2,10,13,14,15].

The composition of wine lees depends on environmental conditions, regions of origin and their agronomic characteristics, the grape variety, and the time of aging in the wood barrels [13,16].

Wine lees are used for wine ageing, usually for white and sparkling wines, although sometimes they are applied in red wines [17]. Wine aging on lees is a traditional oenological technique, which consists in placing wines on their fine lees (essentially dead yeast cells) and some grape solids [18,19]. The autolysis of yeasts, after cell death, leads to the release of cellular proteins, nucleic acids, lipids, and polysaccharides, and provides the conditions for many components within the yeast to leak into the wine [20]. This technique improves the quality of wines because aging on lees reduces the astringency and bitterness and improves the structure and the color stability of wine. In addition, wines are enriched in volatile aromatic compounds [21]. These properties of wine aged on lees seem to be due to the interaction of phenolic compounds with mannoproteins released during yeast lees autolysis [8,19]. The ability of yeast to form molecular interactions with phenolic compounds and adsorb them [22] implies that the wine lees can be considered as a raw source for the extraction of these compounds.

## 3. Phenolic Extraction from Solid Fraction of Wine Lees

The first step for the chemical characterization of the phenolic compounds still present in wine lees is the extraction of these compounds. Extraction is a very important step in the recovery of phenolic compounds, and therefore the extraction parameters must be controlled to obtain extracts rich in phenolics. There is no single extraction method; however the most common technique for extraction of phenolic compounds from wine lees is solid–liquid extraction. The main extraction parameters considered are sample pre-treatment (drying, lyophilization, grinding), solvent, solvent–solid ratio, extraction mode (stirring, ultrasound, microwave), temperature, and time.

**Figure 1:**
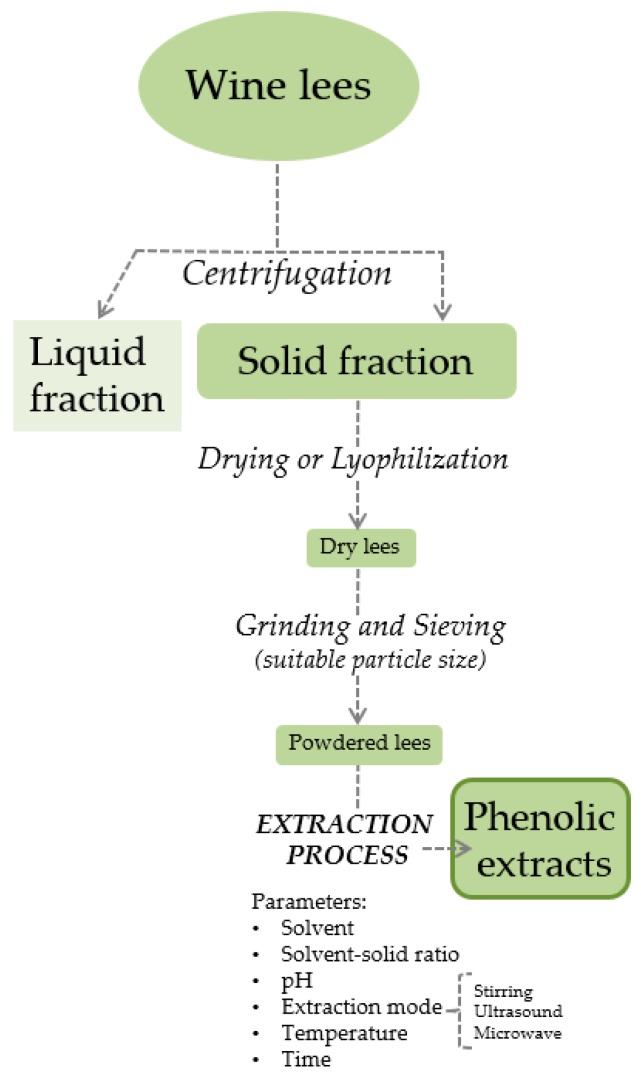
Scheme of the general phenolic extraction process.

Some papers on the phenolic extraction from wine lees found in literature have been summarized in Table 1. In Figure 1, the illustrated process scheme in reference to Table 1 is shown.

Regarding pre-treatment, lees are dried at 40 °C or 50 °C in an oven [19,23,24,25], or lyophilized [2,9,15,26], during 24 or 48 h. As can be observed in Table 1, the most common solvent is ethanol, which is mixed with water in different proportions and sometimes adjusted to acid pH [19,24]. In aqueous solution, anthocyanins are found in various chemical forms with different chromatic properties whose equilibrium depends on the pH. The acidification of the solvent is applied in order to obtain the flavylium cation form (red color), which is stable in a highly acidic medium [27]. Pure water, acetone, and methanol are also used, although these solvents barely extract the phenolic compounds present in the wine lees compared with the mixtures of ethanol:water [9]. According to the referenced study, the ethanol:water mixture corresponding to the ratio 75:25 was the best solvent, with a content of 254 mg gallic acid equivalents (GAE)/g dry lees (DL), while the lowest values were 26 and 38 mg GAE/g DL for acetone and water, respectively. Phenolic extracts obtained from wine lees could be used in the elaboration of foods and pharmaceutical products and therefore it is important to use innocuous solvents to human health. Conventional organic solvents, such as methanol and acetone, have excellent ability of extraction but they are toxic to health and also produce chemical pollution. In this sense, ethanol, water, and their mixtures are the best extraction solvents because they are green solvents and enable direct use in foods and pharmaceutical products.

Regarding the solvent-to-solid ratio parameter, in a study on determination of appropriate ranges of extraction parameters for phenolic compounds from wine lees, the range from 30:1 to 50:1 was selected as the optimal range to obtain the best extraction yield [19].

Bosiljkov et al. (2017) established a highly efficient and eco-friendly extraction method for the anthocyanins in wine lees using natural deep eutectic solvents (NADES). NADES, that are mixtures of choline chloride with a hydrogen donor, coupled with high-efficiency ultrasound-assisted extraction, were an excellent choice for extraction of phenolic compounds from wine lees [26].

Tao et al. (2014) [19] compared the results of ultrasound-assisted extraction and conventional extraction (maceration) and they observed that the extraction yields of total phenolics and total anthocyanins from maceration were 19.8% and 20.5% lower than those from ultrasound-assisted extraction, which indicated that the ultrasound-assisted extraction improves the phenolic extraction from wine lees. Ultrasound technology is used to improve extraction processes because produces heat and mass transfer enhancement due to the acoustic effects and the ultrasonic cavitation phenomenon [29].

Also, microwave-assisted extraction has been used for phenolic extraction from wine lees [24,25]. Perez-Serradilla and Luque de Castro (2011) [24] applied a microwave-assisted extraction for the extraction of phenolic compounds from wine lees and they indicated that this provided a better extraction yield than conventional extraction. In addition, authors reported a shorter extraction time (17 min vs. 24 h).

The time and temperature for phenolic extraction from wine lees is highly variable. The time can vary from 2 min to 3 h; and the temperature from room temperature to 40 °C (Table 1).

## 4. Phenolic Compounds in Wine Lees

Phenolic compounds are transferred from grape to wine during the maceration, but a high proportion of these compounds remain in the winemaking byproducts such as lees. The wine lees contain phenolic compounds due to the adsorption capacity of yeast cell wall [30]. The phenolic profile in lees depends on the type of crushed grapes and other factors that are present during vinification [22].

The total phenolic content of wine lees has been widely evaluated by the Folin–Ciocalteu assay and values were very different between studies. Tao et al. (2014) [19] indicated that the content of total phenolics extracted by ultrasound ranged between 44 and 59 mg GAE/g dry matter (DM), with the final yield at the optimal conditions being 58.77 mg GAE/g DM. However, in other studies lower values were found: 30.86 and 23.16 mg GAE/g DM [28,31]. In the study carried out by Romero-Díez et al. (2018), wines lees were extracted using solvents with different polarities (water, methanol, ethanol, two hydroalcoholic mixtures and acetone), and total phenolic content ranged between 26 and 254 mg GAE/g DM, with the mixture of 75:25 (*v*/*v*) ethanol:water showing the highest efficiency [9]. On the other hand, Perez-Serradilla and Luque de Castro (2011) [24] performed a conventional extraction with 75:25 (*v*/*v*) ethanol:water from wine lees and they reported a total phenolic content of 547 mg GAE/g DM. These differences between results may be due to differences in the types of wine lees (grape variety and vinification process) and mainly the extraction process (solvent and extraction method).

The contents of total non-flavonoids and total flavonoids have been reported: 1332 and 984 mg GAE/100 g DM, respectively. The content of total anthocyanins was also reported: 383 mg of cyanidin-3-glucoside equivalents/100 g of DM [31].

**Figure 2:**
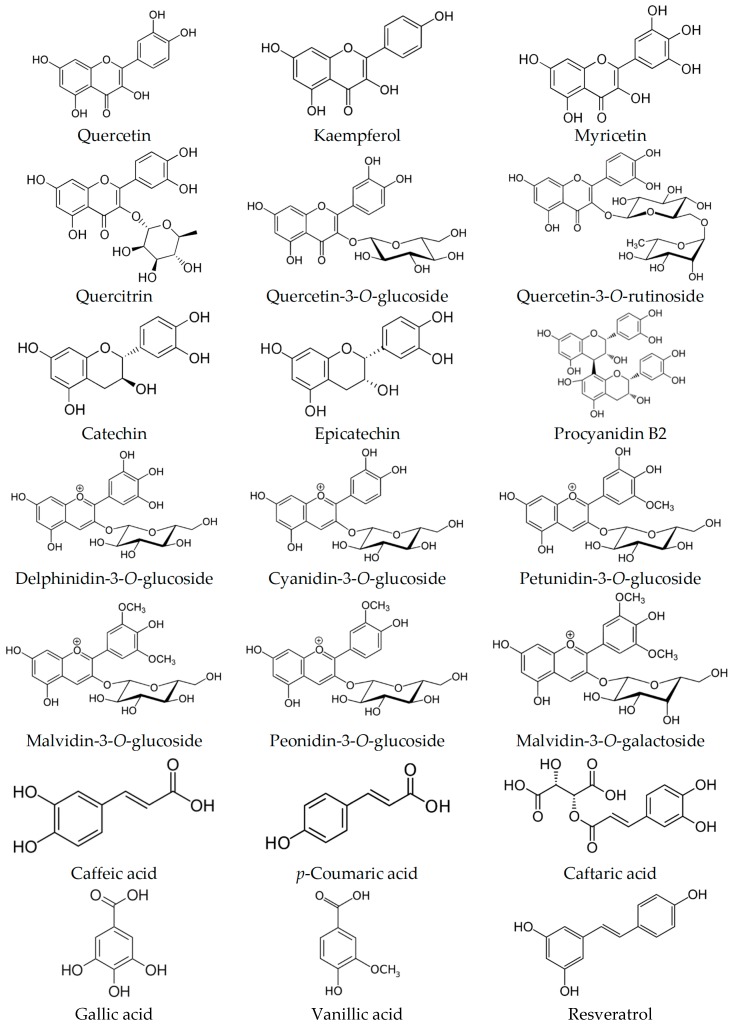
Chemical Structures of some phenolic compounds identified in wine lees.

Individual phenolic compounds belonging to flavonoids (flavanols, flavonols, and anthocyanins), phenolic acids, and stilbenes have been identified and quantified in wine lees. Figure 2 shows some chemical structures belonging to phenolic compounds identified in wine lees. Wine lees are a reliable source of flavonols such as quercetin, quercitrin, kaempferol, and myricetin. According to literature [28], the major flavonol is quercetin with an amount of 42 μg/g DM, while kaempferol and myricetin show lower values (10 and 8 μg/g DM, respectively). Other flavonols detected in wine lees include kaempferol 3-(2′,3′-diacetylrhamnoside)-7″-rhamnoside, quercetin 3-*O*-glucoside, quercetin 3-*O*-glucuronide, quercetin 3-*O*-galactoside and quercetin 3-*O*-rutinoside [2,25,32]. In wine lees from *Vitis labrusca* varieties, laricitrin, isorhamnetin, and syringetin, and their glucosides, as well as myricetin derivatives (myricetin 3-*O*-glucuronide and myricetin 3-*O*-glucoside) were found [2].

In a paper on characterization of wine lees by liquid chromatography and mass spectrometry, flavanols, namely catechin, epicatechin, and procyanidin B2, were tentatively identified, but these compounds were not quantified [25]. In other studies, the content of catechin was 4 μg/g DM [28] and 121 μg/mL wine lees extract [32]. These concentrations were low in comparison with other compounds present in the samples, such as quercetin (42 μg/g DM and 1216 μg/mL wine lees extract, respectively).

Anthocyanins have been identified in wine lees. Up to total of 26 anthocyanins were determined in samples of wine lees: derivates of the anthocyanidins delphinidin, cyanidin, petunidin, peonidin and malvidin. In *Vitis vinifera* varieties, delphinidin-3-*O*-glucoside, petunidin-3-*O*-glucoside, peonidin-3-*O*-glucoside, malvidin-3-*O*-glucoside, malvidin-3-*O*-galactoside, delphinidin-3-*O*-(6″-*p*-acetylglucoside), cyanidin-3-*O*-(6″-*p*-acetylglucoside), malvidin-3-*O*-(6″-*p*-acetylglucoside), delphinidin-3-*O*-(6″-*p*-coumaroyl-glucoside), petunidin-3-*O*-(6″-*p*-coumaroyl-glucoside), malvidin-3-*O*-(6″-*p*-coumaroyl-glucoside) and pelargonidin-3-(6″-*p*-coumaryl-glucoside) were found [9,25,33]. Among these compounds, malvidin-3-*O*-glucoside and malvidin-3-(6″-*p*-coumarylglucoside) were in higher concentrations in wine lees [9].

On the other hand, the anthocyanin profile of such by-products from two *non-vinifera* Brazilian grape varieties was dominated by diglucoside derivatives in two main forms: 3,5-diglucosides and their *p*-coumaroylated derivatives (3-(6″-coumaroyl)-glucoside-5-glucosides) of the anthocyanidins previously mentioned [2].

Regarding phenolic acids, caffeic acid and *p*-coumaric acid and their derivates such as *trans*-caftaric acid and *trans*-coutaric acid, respectively, have been found in wine lees samples, being caffeic acid and *trans*-caftaric acid the predominant compounds in both *Vitis vinifera* and non-vinifera varieties [2,28]. Also, hydroxybenzoic acids have been reported: gallic and vanillic acids [25,28]. All acids were found in concentrations ranging between 1 and 6 μg/g DM, for gallic acid and caffeic acid, respectively.

Resveratrol, *cis*- and *trans*-resveratrol, is a stilbene that has been extensively identified in skin of grapes, however its identification in wine lees is less common. This compound has been reported by some authors but at lower concentrations than other phenolics [2,32].

## 5. Antioxidant Activity

Several pathologies, health conditions and degenerative processes such as atherosclerosis, diabetes, arterial hypertension, cancer, and aging are related to the oxidative stress. Oxidative stress generates an important cell damage because to a high production of reactive oxygen species (ROS), which attack macromolecules such as proteins, DNA, and lipids. Phenolic compounds in wine lees show different antioxidant activities for different assays and therefore lees are a good source of antioxidant compounds that can delay the oxidation of macromolecules [9].

The antioxidant activity of wine lees has been determined using different methodologies such as 3-ethylbenzothiazoline-6-sulfonic acid (ABTS), 1,1-diphenyl-2-picrylhydrazyl (DPPH), and oxygen radical absorbance capacity (ORAC) assays that measure the ability of antioxidants to scavenge a radical [9,34,35]. The radical scavenging activity has been also measured by the generation of three different free radicals [28]: hydroxyl radical scavenging activity based on the generation of HO• by the Fenton reaction, peroxyl radical (ROO•) scavenging activity based on the thermic decomposition of 2,4-dichlorofluoroscein, and the scavenging activity against superoxide anion (O2•^−^) based on a hypoxanthine (HPX)/xanthine oxidase (XOD) system. Also, antioxidant activity of wine lees has been determined by cupric reducing antioxidant capacity (FRAP) assay that measures the capacity of antioxidants to reduce the Fe (III) complex of 2,4,6-tripyridyl-s-trizin (TPTZ) to Fe (II)- TPTZ chelate [31].

Usually, antioxidant activity from winemaking byproducts is directly related to the total concentration of phenolic compounds, being the highest total phenolic content values, which correspond with the highest antioxidant activity values [36]. Regarding wine lees, wine polyphenols retained by lees contribute to the antioxidant effect [35,37]. According to literature, antioxidant activity values range between 200 and 6000 μmoL Trolox equivalents (TE) per gram of DL, depending on the extraction method and the antioxidant activity assay [9,24]. The phenolic extracts obtained with ethanol:water mixtures (especially the 75:25 EtOH:H_2_O (*v*/*v*) mixture) have higher antioxidant capacities than the rest of the extracts (water, ethanol, acetone and methanol).

Compounds contributing to antioxidant activity have been studied, and it has been established which compounds or group contribute to each assay: flavanols showed negative correlations with ORAC and positive with FRAP assays; specifically, catechin showed statistically significant positive correlation with FRAP and with hydroxyl radical scavenging capacity (HOSC) assays. Flavonols showed different correlations with ORAC, FRAP, hydroxyl radical averting capacity (HORAC), and HOSC. The correlations were significant and positive for quercetin-3-*O*-glucuronide with ORAC and HORAC, and for myricetin with HOSC and FRAP. Regarding anthocyanins, for ORAC and HORAC the correlation was negative and for HOSC and FRAP was positive [9].

Landeka et al. (2017) determined the antioxidant capacity of wine lees by the DPPH and FRAP methods and results were 259 and 45 mM TE/100 g DL for DPPH and FRAP, respectively. In addition, in this study, wine lees had hypolipidemic and antioxidant properties in animal model [31].

However, wine lees show scavenging activity lower than that found in grape pomace [28], which could be due to a lower total phenolic content of lees respect to grape pomace (90.21 mg GAE/g vs 30.86 mg GAE/g DM).

Although phenolic compounds are the main compounds contributing to the antioxidant activity, recently another high-value chemical compound has been recovered from wine lees: squalene, which is a natural antioxidant synthesized during sterol biosynthesis in plants [38].

On the other hand, the antioxidant properties of wine lees are also important because they can be used for the elaboration of sparkling wines. The ageing on lees of wines has a close relationship with their phenolic profile [39].

## 6. Conclusions

Wine lees are winemaking byproducts with high content of antioxidant compounds, consisting of mainly phenolic compounds. Flavonols and anthocyanins (in wine lees from red grapes) are the most abundant phenolic compounds in wine lees. Further studies are required to determinate other components present in wine lees which could contribute to these antioxidant effects.

The exploitation for the potential reuse of wine lees in the wine industry or other industries could be of great interest due to their chemical composition and antioxidant properties.

## Figures and Tables

**Table 1:** Phenolic extraction from wine lees samples.

Sample	Pre-Treatment	Solvents	Extraction Mode	Reference
Wine lees from red grapes (*Vitis vinifera* cv. Syrah).	Lees were centrifuged at 2100× *g* and the solid phase was dried at 40 °C for 48 h in an oven, milled, and sieved (particle size: 0.5 mm).	The lees were mixed with ethanol 75% (hydrochloric acid 1% in water) in a 1:10 (*w*/*v*) ratio.	(1) A microwave-assisted extraction at 200 W irradiation power for 17 min was applied. (2) The mixture was stirred at 40 °C for 24 h.	[24]
Wine lees from red grapes (*Vitis labrusca* hybrid varieties: BRS Violeta and BRS Lorena).	Lees were freeze-dried for 48 h.	A sample of 0.25 g was extracted with 50 mL of methanol/water/formic acid (50:48.5:1.5, *v*/*v*/*v*).	The mixture was placed in ultrasonic bath during 2 min and centrifuged at 5000× *g* at 5 °C for 5 min.	[2]
Wine lees from red grapes (mixture of *Vitis vinifera* cv. Cabernet Sauvignon 60%, Merlot 30%, and Cabernet Franc 10%).	Wine lees were dried in an oven at 40 °C for 48 h, and then milled and sieved (particle size: smaller than 0.6 mm).	Dried wine lees and 50 mL of aqueous ethanol solution were mixed.	(1) The mixture was placed in an ultrasonic bath system and centrifuged at 12,000 rpm for 10 min. (2) Conventional solvent extraction: maceration.	[19]
Wine lees from red grapes (variety not mentioned).	Lees were dried in a climate chamber at 40 °C, ground, and sieved (particle size: 100–300 µm).	A sample of 1 g was extracted with 25 mL ethanol/water (1:1).	The mixture was placed in an ultrasound bath.	[23]
Wine lees from red grapes (*Vitis vinifera* cv. Tempranillo, Merlot, Garnacha, Cabernet, and Mazuelo).	Lees were centrifuged at 855× *g* and the solid phase was dried at 40 °C for 48 h in an oven, milled, and sieved (particle size: 0.5 mm).	A sample of 6 g was mixed with 50 mL of 60:40 (*v*/*v*) ethanol-water (adjusted to pH 4 with formic acid).	The mixture was placed in a microwave-assisted digestor at 140 W irradiation power for 10 min.	[25]
Wine lees from white grapes (*Vitis labrusca* cv. Niagara).	Lees were freeze-dried.	A sample of 1 g was homogenized with 3 mL of pure water.	The mixture was agitated at 150 rpm overnight at room temperature.	[15]
Wine lees from red grapes (*Vitis vinifera* cv. Pinot noir).	Lees were dried in an air-circulation oven for 12 h at 50 °C and ground.	A sample of 20 g was homogenized with 150 mL of ethanol/water/formic acid (50:48.5:1.5, *v*/*v*).	The mixture was placed in a blender for 2 min and centrifuged at 2500× *g* for 15 min.	[28]
Wine lees from red grapes (*Vitis vinifera* cv. Merlot).	Lees were lyophilized.	(1) NADES: mixtures of choline chloride with a hydrogen donor. (2) Ethanol/water/formic acid (50:48.5:1.5, *v*/*v*/*v*) at pH 2.7.	The mixtures were placed in an ultrasonic bath system with different time and ultrasonic power depending on an experimental design.	[26]
Aging wine lees from red grapes (*Vitis vinifera* cv. Tempranillo).	Lees were centrifuged for 90 min at 10,000 rpm and were freeze-dried for 48 h.	A sample of 0.25 g was mixed in 10 mL of solvent: distilled water, ethanol, acetone, methanol and two mixtures of ethanol:water (50:50 and 75:25 *v*/*v*).	The mixture was stirred for 5 min at room temperature followed by 10 min of sonication in an Transsonic 700/H bath.	[9]

## Abbreviations

ABTS	3-ethylbenzothiazoline-6-sulfonic acid
DL	dry lees
DM	dry matter
DPPH	1,1-diphenyl-2-picrylhydrazyl
FRAP	cupric reducing antioxidant capacity
GAE	gallic acid equivalents
HORAC	hydroxyl radical averting capacity
HOSC	hydroxyl radical scavenging capacity
NADES	natural deep eutectic solvents
ORAC	oxygen radical absorbance capacity
TE	Trolox equivalents
TPTZ	2,4,6-tripyridyl-s-trizin
